# 703 Evaluation of a Stress Ulcer Prophylaxis Protocol in an Burn Intensive Care Unit

**DOI:** 10.1093/jbcr/irac012.257

**Published:** 2022-03-23

**Authors:** Zachary Drabick, Ian Driscoll, Duong Nguyen

**Affiliations:** UF Health Shands Burn Center, High Springs, Florida; UF Health Shands Burn Center, Gainesville, Florida; Texas Children’s Hospital, Houston, Texas

## Abstract

**Introduction:**

Patients suffering from large burn injuries have an increased risk of gastrointestinal mucosal damage from stress ulcers. Proton pump inhibitors (PPIs) and histamine H2-receptor antagonists (H2RAs) are routinely used for stress ulcer prophylaxis in burn patients, however there are few comparative trials evaluating the efficacy of these therapies in the burn population. Our burn center uses a stress ulcer prophylaxis protocol that incorporates both classes in a step-wise manner. The objective of this study was to evaluate the protocol by comparing the incidence of uncontrolled gastric pH, positive gastric occult blood, and gastrointestinal bleed (GIB) while on famotidine or pantoprazole prophylaxis.

**Methods:**

This was a single-center retrospective observational study conducted at an academic medical center and was approved by the hospital’s Institutional Review Board. Patients were included if they were at least 18 years of age, admitted to the burn intensive care unit (BICU) from June 2017 to August 2020 with burns involving at least 1% TBSA, and had at least one documented gastric pH and/or occult blood test while receiving famotidine or pantoprazole. The primary endpoint was a composite of gastric pH less than 5, a positive occult blood test, or occurrence of GIB.

**Results:**

In total, 107 patients with a mean age of 55 yo were included in the study. The median TBSA burn was 16% and length of stay was 23 days. Seventy seven (72%) patients received famotidine and 93 (87%) of patients received pantoprazole. The incidence of the primary endpoint in all patients receiving famotidine and pantoprazole were 69 (90%) and 86 (92%), respectively (*p* = 0.513). During famotidine prophylaxis, 256 (43%) gastric pH tests were less than 5 and 397 (47%) gastric occult blood tests were positive, with no reports of GI bleeding. During pantoprazole prophylaxis, 751 (33%) gastric pH tests were less than 5 and 1220 (47%) gastric occult blood tests were positive, and 4 (4%) were diagnosed with a GI bleed. A total of 91 (85%) patients had deviations from the protocol, which included 42 (46%) patients who did not receive the recommended dose or agent, 27 (30%) patients who were not initiated on the recommended initial agent, and 22 (24%) patients who did not receive a recommended increase in dose or switch agents when recommended.

**Conclusions:**

In the setting of burn injuries of at least 1% TBSA, no difference was detected in uncontrolled gastric pH, positive occult blood tests, or GIB when using famotidine or pantoprazole in our stress ulcer prophylaxis protocol. The overall incidence of gastric pH less than 5 and positive gastric occult blood was high for both agents, while the incidence of GIB was low in both groups.

